# Nanofiber network with adjustable nanostructure controlled by PVP content for an excellent microwave absorption

**DOI:** 10.1038/s41598-019-38899-8

**Published:** 2019-03-12

**Authors:** Jing Lv, Weihua Gu, Xiaoqing Cui, Sisi Dai, Baoshan Zhang, Guangbin Ji

**Affiliations:** 1 School of Electronic Science and Engineering, Nanjing University, Nanjing, 210093 P.R. China; 2 College of Materials Science and Technology, Nanjing University of Aeronautics and Astronautics, Nanjing, 210016 P.R. China

## Abstract

Carbon nanofibers were widely utilized to improve microwave absorption properties since they are a promising lightweight candidate. Adjustable conductive nanostructures of carbon nanofibers were synthesized by electrospinning technique. The conductive network is controlled by the polyvinyl pyrrolidone (PVP) content due to the special hygroscopicity of PVP. The increased adhesive contacts of nanofibers provide more transmission paths for electrons to reduce the effect of air dielectric. Satisfactorily, the carbon nanofibers that carbonized from the polyacrylonitrile (PAN) and PVP (the mass ratio is 6:4) show excellent microwave absorption performance. The minimum reflection loss (RL) value is −51.3 dB at 15.2 GHz and the maximum effective absorption frequency width (<−10 dB) is 5.1 GHz with the matching thickness of only 1.8 mm. Thereby, we believe that this research may offer an effective way to synthesize lightweight carbon nanofibers microwave absorbents.

## Introduction

In modern society, electromagnetic (EM) absorbents have become an indispensable part of information equipment, which can reduce the harm to the surroundings and the humans. The principle of EM absorbers is making microwave transform into other types of energy to attenuate EM wave^1–3^. Traditional microwave absorption materials like metal as well as alloys, ferrites and all varieties of these composites with strong microwave absorption abilities and board effective frequency width develop rapidly. However, it is also significant for actual demand to reduce the density and weight of microwave absorbers at the same time^4–6^.

As famous lightweight materials, carbon nanomaterials have been achieved tremendous attention. Various classifies of carbon nanoparticles such as carbon nanotubes, carbon cloths and so on are of many wonderful characters such as large areas, low cost, good stability and great electrical conductivity^7–10^. Thereby, carbon nanomaterials are quite befitting for EM wave absorption field^11,12^. Among numerous carbon nanomaterials, carbon nanofibers are of huge interest for EM wave absorption due to their one-dimensional nanostructure that can form conductive network^13–15^. For instance, Liu *et al*. successfully fabricated a kind of helical CNFs coated-carbon fibers through catalytic chemical vapor deposition. The minimum RL value was −32 dB at 9.0 GHz and the widest effective frequency width was 9.8 GHz with only 15% filler ratio^16^. Porous carbon nanotubes decorated carbon nanofibers were also achieved with the minimum RL value of −44.5 dB at 10.7 GHz as well as the broad effective frequency width of 7.1 GHz^17^. Chu *et al*. compared the microwave absorption abilities of different diameters, they believed that complex permittivity improved along with the decreasing diameters since it contributed to the conductive network^18^. Based on their researches, we can discover that the microwave absorption performance closely depends on their design of one-dimensional nanostructures on carbon nanofibers. In principle, morphology change of carbon nanofibers could dramatically influence the transferring path of the electrons as well as the construction of the conductive network^19^. Accordingly, the permittivity and polarization process would be controlled in the range of testing frequency artificially. Furthermore, electrospinning fiber technique can be used accurately to obtain diversiform nanofibers, which is a good choice to synthesize carbon nanofibers^20,21^.

In this paper, we added PVP as a structural adjuster to modify PAN based carbon nanofibers by changing the PVP content, followed by electrospinning and next annealing process. PVP, as a high-molecular compound, it is liable to dissolve in water. In addition, the more average molecular weight of PVP, the more chance of it to be agglutinating^22^. Considering with these intrinsic qualities of PVP, we easily gain nanofiber network with adjustable nanostructure controlled by PVP content. Gratifyingly, the microwave absorption performance is enhanced, too. For the carbon nanofibers carbonized from the PAN and PVP (the mass ratio is 6:4 and the filler ratio is 20%), the RL value is −51.3 dB at 15.2 GHz and the maximum effective absorption frequency width (<−10 dB) is 5.1 GHz with only 1.8 mm. This work provides a novel strategy to build the conductive network of carbon nanofibers through adjusting the content of PVP, which may be impulse the development of the lightweight microwave absorbers.

## Results

**Figure 1:**
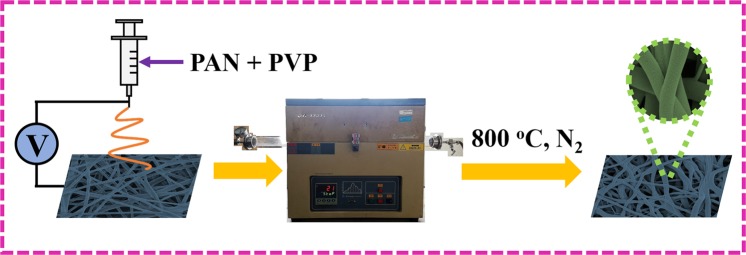
Synthesis schematic diagram of carbon nanofibers with adjustable nanostructure.

Figure 1 is the entire synthesis schematic diagram of our nanofibers conductive network. The precursor solution of PAN and PVP was transformed into nanofibers via a representative electrospinning technology. After being treated at 800 °C for 3 h under N_2_ atmosphere inside tube furnace, carbon nanofibers with adjustable nanostructure were finally fabricated.

**Figure 2:**
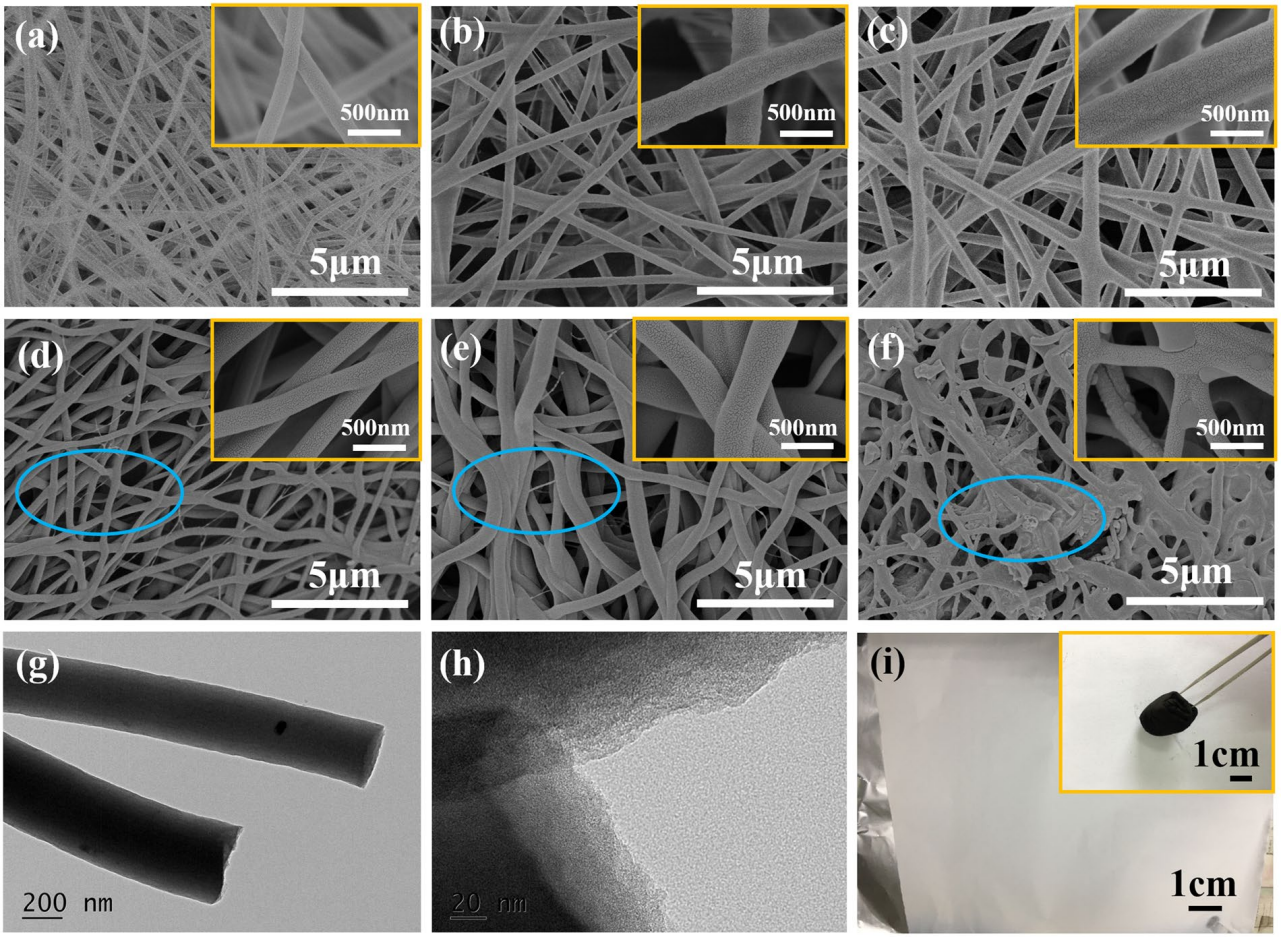
SEM images of (**a**) PAN/PVP-7/3 (**b**) PAN/PVP-6/4 (**c**) PAN/PVP-5/5 (**d**) PVP (214) (**e**) PVP (333) (**f**) PVP (500) samples, Inserts are magnified images; (**g**,**h**) TEM images of PVP (333) sample; (**i**) physical photo of PAN/PVP-6/4 sample, Insert is the physical photo of PVP (333) sample.

Detailed nanostructures of different nanofibers are detected using SEM and TEM methods. Obviously, nanofibers of PAN/PVP samples show typical one-dimensional morphology of electrospun fibers in Fig. 2(a–c). There surfaces are smooth. In addition, we can easily see that the nanofibers become more and more thicker since the quality of PAN is constant and the quality of PVP is larger. The average diameters of PAN/PVP-7/3 sample, PAN/PVP-6/4 sample and PAN/PVP-5/5 sample are 167 nm, 277 nm and 444 nm, respectively. After calcined at 800 °C under N_2_ atmosphere, it can be seen that some of nanofibers are bent and bonded in Fig. 2(d–f). Notably, this phenomenon is relatively clearer with the increasing content of PVP. Due to the strong hygroscopicity of PVP, the solvent doesn’t volatilize very well so that the nanofibers change into a sticky one. However, nanofibers of PVP (500) sample present a fracture situation. It is very difficult to identify the one-dimensional nanofibers in Fig. 1(f). Moreover, Fig. 2(g,h) displays the TEM pictures of PVP (333) sample. It should be noticed that there is typical carbon margin in Fig. 2(h). Also, physical photos of the white PAN/PVP-6/4 sample and the black PVP (333) sample are provided in Fig. 2(i).

**Figure 3:**
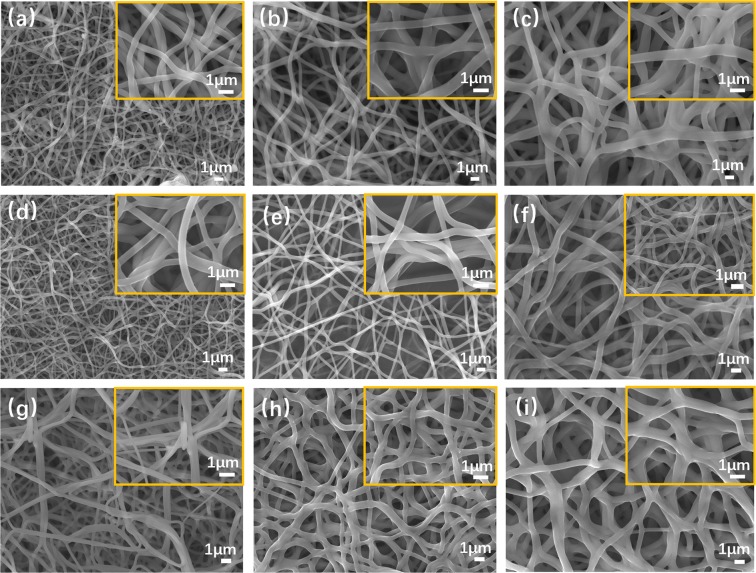
SEM images of (**a**) PVP (214–200) (**b**) PVP (333–200) (**c**) PVP (500–200) (**d**) PVP (214–400) (**e**) PVP (333–400) (**f**) PVP (500–400) (**g**) PVP (214–600) (**h**) PVP (333–600) (**i**) PVP (500–600) samples. Inserts are magnified images.

The PAN/PVP nanofibers were also heated at 200 °C, 400 °C and 600 °C. As can be seen from Fig. 3, all of these samples present a curved one-dimensional nanofibers morphology. The nanofibers are getting thicker with the increasing content of PVP. Besides, the fusing phenomenon become serious when we added more PVP.

**Figure 4:**
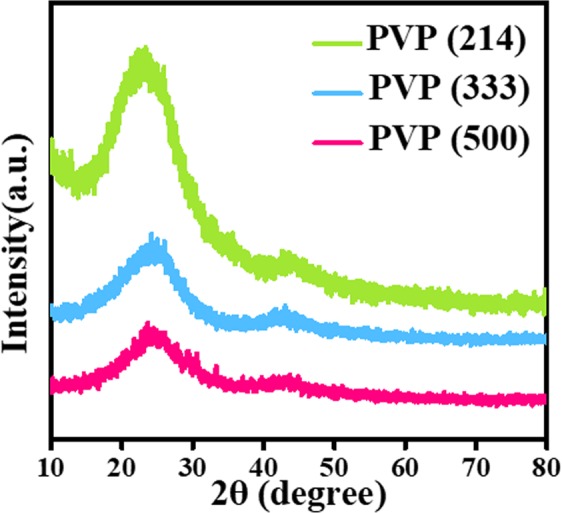
XRD patterns of PVP (214), PVP (333) and PVP (500).

Figure 4 offers us with the similar XRD patterns of PVP (214) sample, PVP (333) sample and PVP (500) sample. Obviously, we have successfully achieved amorphous carbon nanofibers. No impurities can be detected.

**Figure 5:**
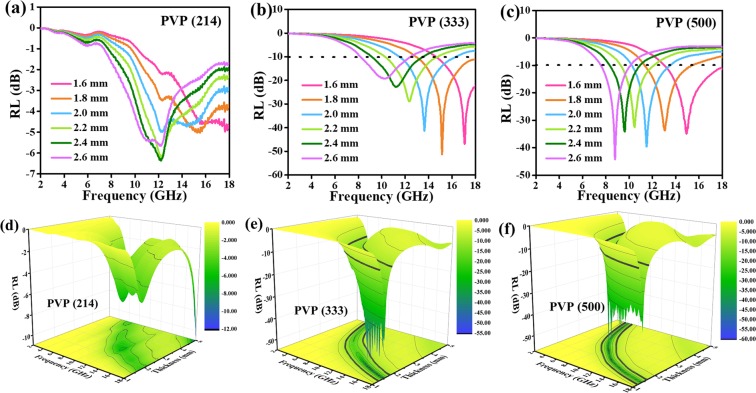
RL values of (**a**) PVP (214) (**b**) PVP (333) and (**c**) PVP (500) samples with matching thickness of 1.6 mm, 1.8 mm, 2.0 mm, 2.2 mm, 2.4 mm and 2.6 mm; 3D RL plots of (**d**) PVP (214) (**e**) PVP (333) (**f**) PVP (500) samples in the range of 2–18 GHz.

**Figure 6:**
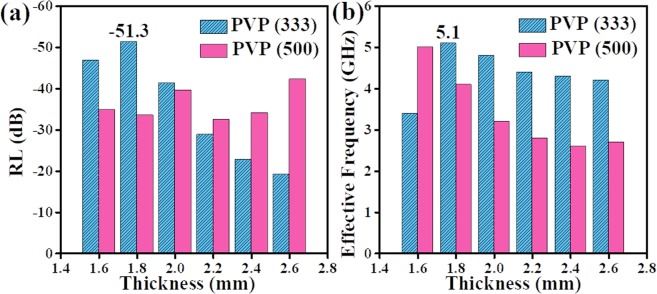
(**a**) RL peak values and (**b**) *f*_E_ peak values of PVP (333) sample and PVP (500) sample with different thickness of 1.6 mm, 1.8 mm, 2.0 mm, 2.2 mm, 2.4 mm and 2.6 mm.

The RL values of different carbon nanofibers are provided in Fig. 5(a–f) using the following formulas^23–25^:

1 $$RL=20\,{\mathrm{log}}_{10}|({Z}_{in}-{Z}_{{0}})/({Z}_{in}+{Z}_{{0}})|$$

2 $${Z}_{in}=\surd ({\mu }_{r}/{\varepsilon }_{r}){\tanh }[j(\frac{2\pi }{c})\,fd\,\surd ({\mu }_{r}{\varepsilon }_{r})]$$

where *Z*_*in*_ is the input impedance, *Z*_0_ is the impedance of free space, *f* is the frequency, *d* and *c* represent the thickness and the velocity of light, respectively. When the thickness increases, the RL values shifts to lower frequency, indicating that EM wave absorption frequency and the thickness of absorbers can be modulated. Among PVP (214), PVP (333) and PVP (500) samples, PVP (214) sample shows the poorest microwave absorption abilities. Different microwave absorption performances have something to do with the content of PVP. As the RL values below −10 dB, effective microwave absorbers will make 90% microwave attenuated^26^. Thereby, when matching thickness are 1.6 mm, 1.8 mm, 2.0 mm, 2.2 mm, 2.4 mm and 2.6 mm, RL values of PVP (333) sample and PVP (500) sample can meet the actual application requirements. That is, for PVP (333) sample, the minimum RL value is −51.3 dB at 15.2 GHz and the maximum effective absorption frequency width (*f*_E_, < −10 dB) is 5.1 GHz both with 1.8 mm of thickness. Moreover, for PVP (500) sample, the minimum RL value is −44.3 dB at 8.8 GHz with 2.6 mm as well as the maximum effective absorption frequency width is 5.0 GHz with only 1.6 mm. To compare the microwave absorption performance of PVP (333) sample and PVP (500) sample, RL peak values and *f*_E_ peak values of PVP (333) and PVP (500) with different thickness are given in Fig. 6(a,b). As seen in Fig. 6, PVP (333) sample has more boarder effective frequency width while the microwave absorption ability of PVP (500) sample is stronger.

**Figure 7:**
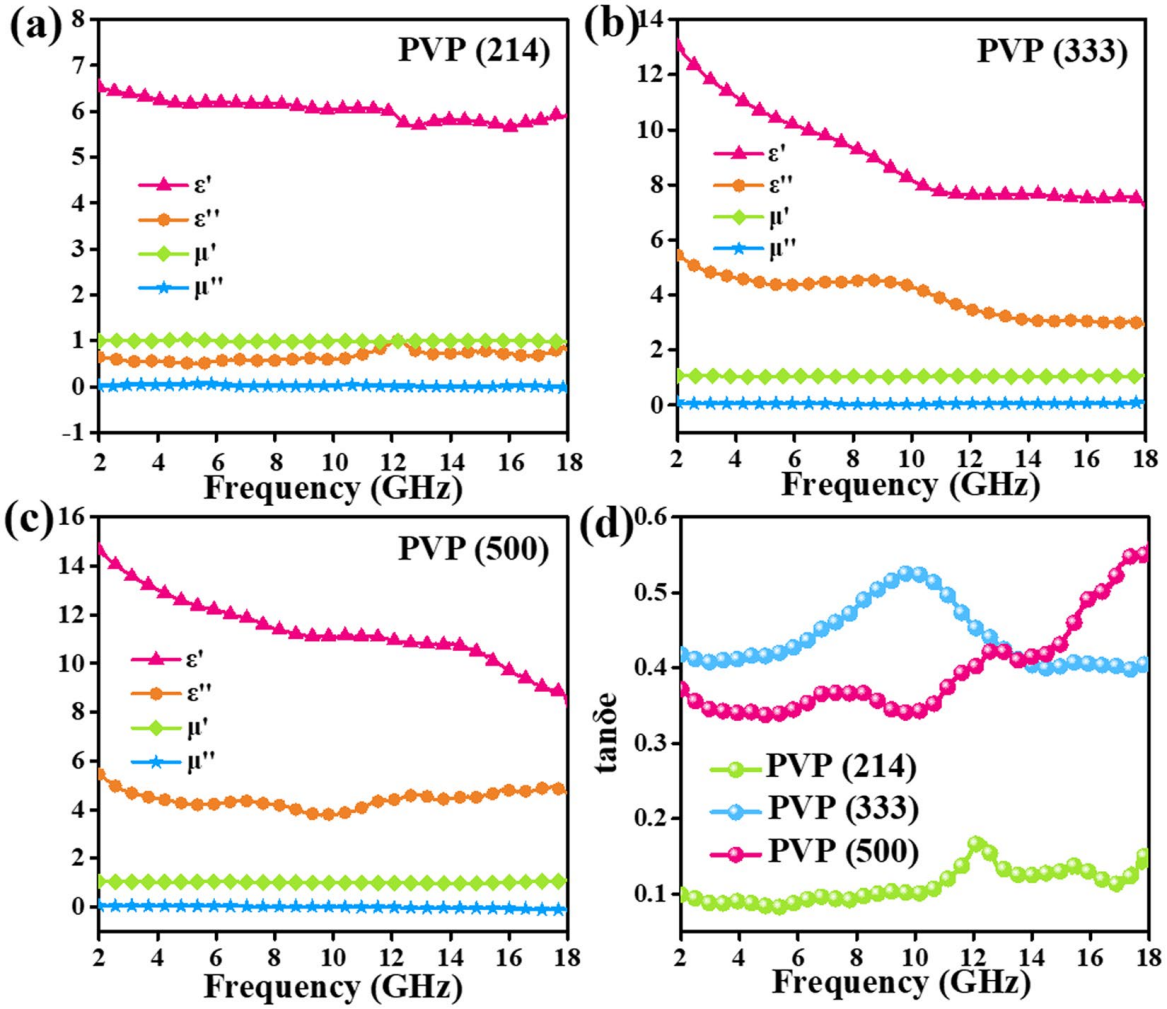
Frequency dependence on electromagnetic parameters of (**a**) PVP (214) (**b**) PVP (333) and (**c**) PVP (500) samples in the range of 2–18 GHz.

The electromagnetic parameters (complex permittivity: ε = ε′ - jε″; the complex permeability: μ = μ′ - jμ″) of carbon nanofibers are tested by a network analyzer. Herein, magnetic properties of carbon nanofibers can be neglected since the dielectric loss more critical. Obviously, permittivity shows a sustainable growth with the increasing content of PVP. The ε′ of PVP (214) sample is the lowest among these carbon nanofibers. The ε′ of PVP (333) sample is from 13.0 to 7.2 while PVP (500) sample descends from 14.6 to 8.2. The ε″ values of PVP (333) sample and PVP (500) sample are higher than that of PVP (214) sample. In Fig. 7, some small fluctuations of ε″ curves arise from the multiple nature resonances. According to pervious reports, it was found that the complex permittivity would improve with the increasing average diameters of the PAN based carbon nanofibers^18^. Nevertheless, our nanofibers do not suit this situation. This phenomenon may refer to their special adhesive nanostructures. Namely, average diameters could not be the main reason that effects the complex permittivity. As we all know, if there are some gaps between nanofibers, air will reduce the dielectric of nanofibers, bringing about lower permittivity values. Therefore, the adhesive carbon nanofibers would be the dominant reason that increases the dielectric constant. Thanks to the function of PVP, the more contact carbon nanofibers, the electrical conductivity is higher. As studied by other groups, dielectric loss can be caused by conductive loss as well as by polarization loss involved in the relaxation process^27^. Moreover, different polarization process including ionic polarization, electronic polarization and dipole polarization can be considered in this cause^28^. However, ionic polarization and electronic polarization always happen at high frequency such as 10^3^–10^6^ GHz, dipole polarization should be the main reason that leads to the polarization process. Using the Eq. (3), dielectric loss degree can be evaluated in Fig. 7(d)^29–31^:

3 $$\tan \,{{\rm{\delta }}}_{{\rm{e}}}={\rm{\varepsilon }}^{\prime\prime} /{\rm{\varepsilon }}^{\prime} $$

The results manifest that the dielectric loss tangent of PVP (214) sample is around 0.1. The dielectric loss tangent curve of PVP (333) sample show a rising trend followed by a drop condition. PVP (500) sample show a raising trend. So, we suggest that both PVP (333) sample and PVP (500) are of higher dielectric loss.

**Figure 8:**
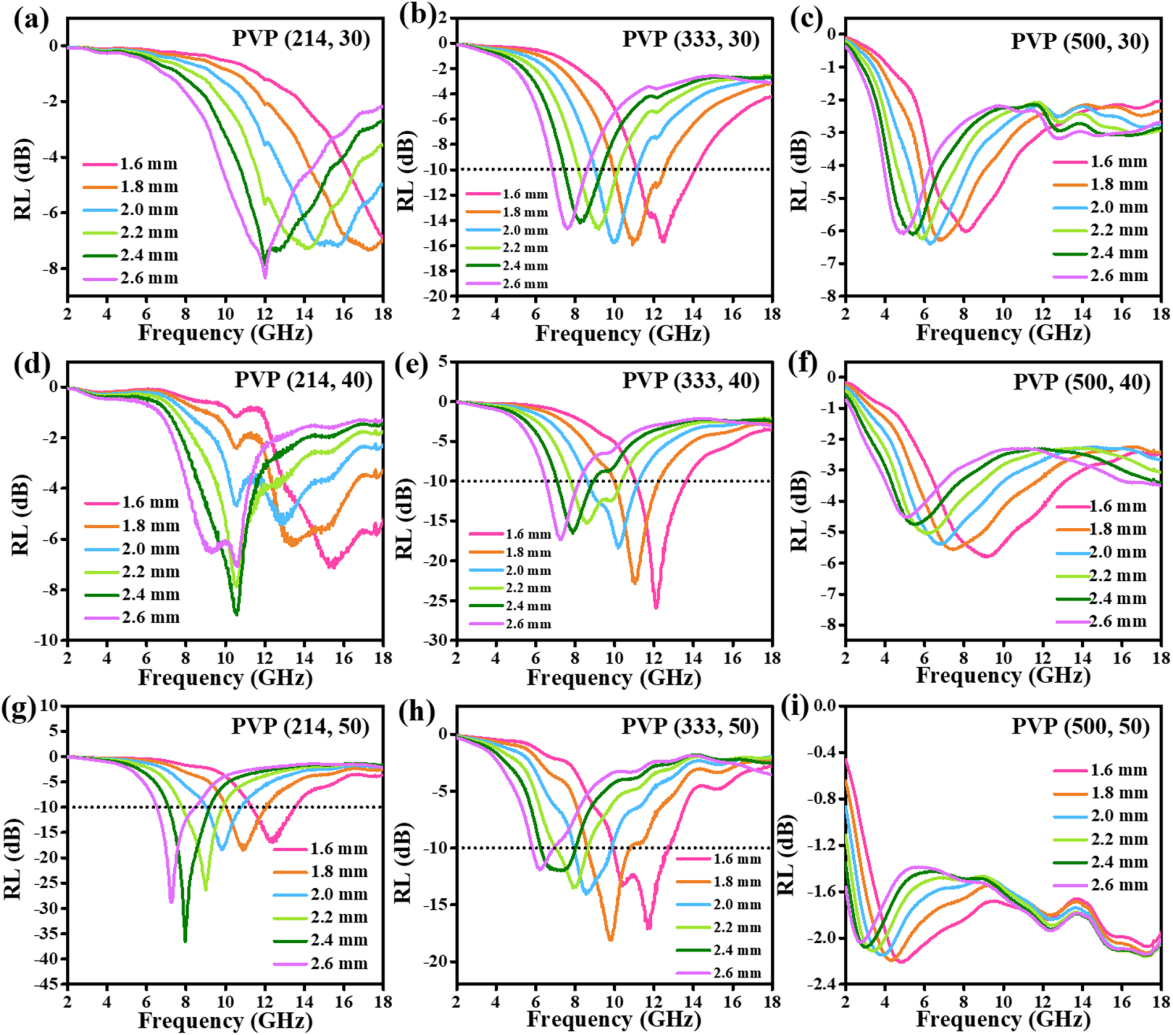
RL values of (**a**) PVP (214, 30) (**b**) PVP (333, 30) (**c**) PVP (500, 30) (**d**) PVP (214, 40) (**e**) PVP (333, 40) (**f**) PVP (500, 40) (**g**) PVP (214,50) (**h**) PVP (333, 50) and (**i**) PVP (500, 50) in range of the 2–18 GHz.

**Figure 9:**
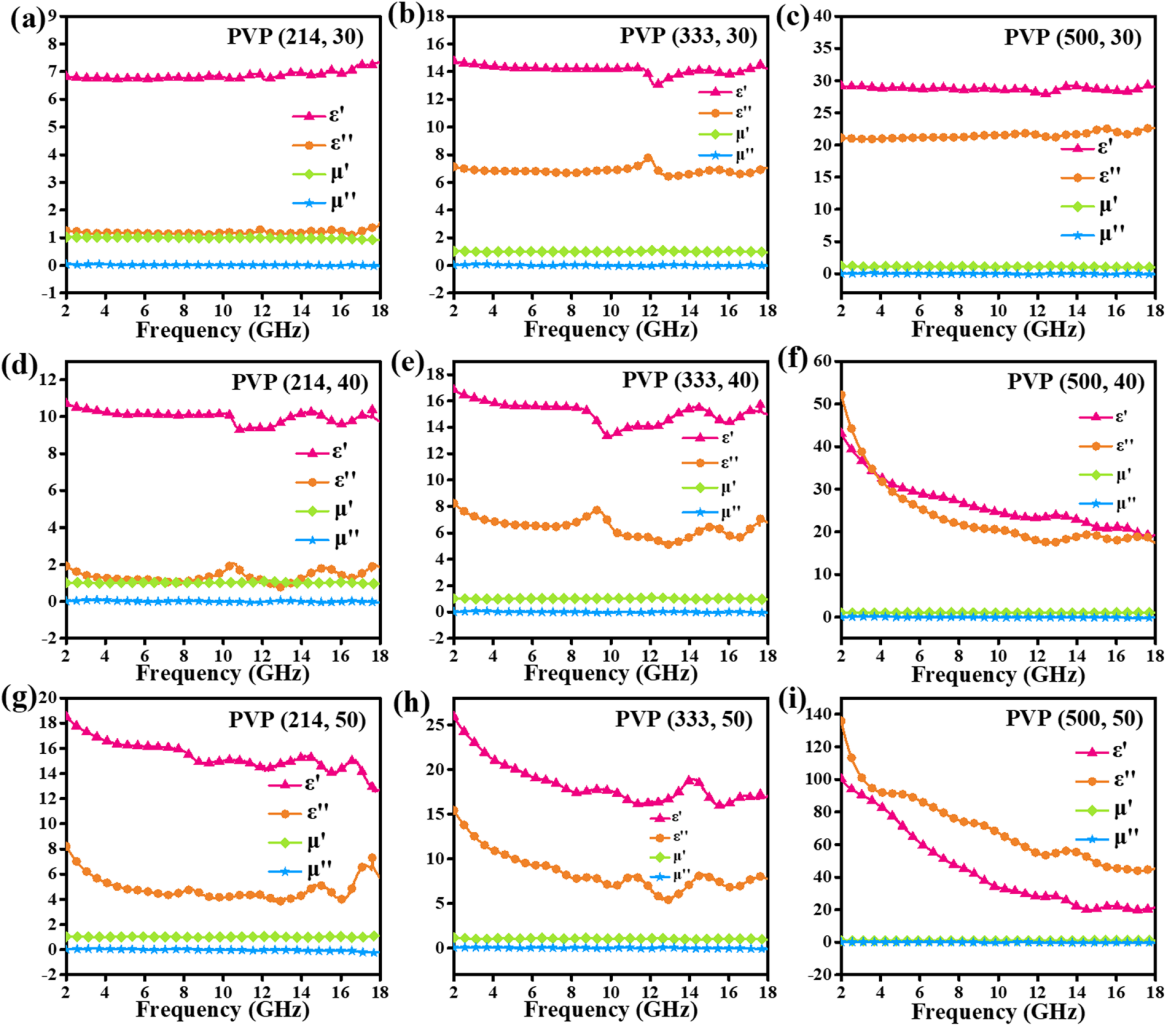
Frequency dependence on electromagnetic parameters of (**a**) PVP (214, 30) (**b**) PVP (333, 30) (**c**) PVP (500, 30) (**d**) PVP (214, 40) (**e**) PVP (333, 40) (**f**) PVP (500, 40) (**g**) PVP (214,50) (**h**) PVP (333, 50) and (**i**) PVP (500, 50).

The RL values and frequency dependence on electromagnetic parameters of different sample filler ratios are displayed in Figs 8 and 9. Accordingly, the permittivity goes up with the increasing sample filler ratio. When the sample filler ratios are 30% and 40%, the microwave absorption abilities of PVP (333) sample are much better than that of other two samples. Interestingly, RL values decreases with the increasing content of PVP when the sample filler ratio is 50%. Particularly, PVP (214) shows the minimum RL value of −36.5 dB at 8.0 GHz with the thickness of 2.4 mm.

**Figure 10:**
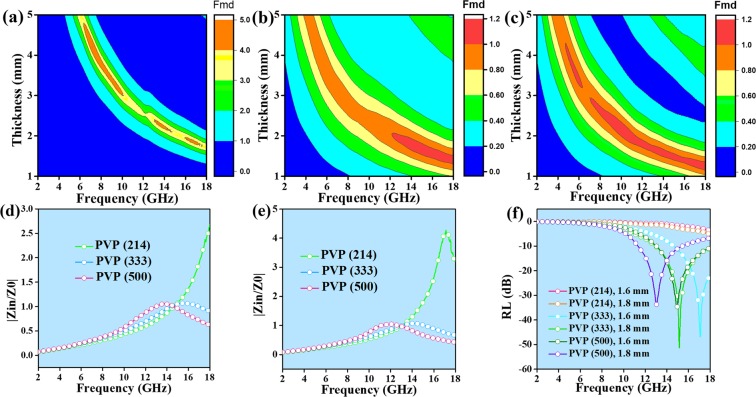
3D representation of |Z_in_/Z_0_| values of (**a**) PVP (214) (**b**) PVP (333) (**c**) PVP (500) samples; |Z_in_/Z_0_| values of PVP (214), PVP (333) and PVP (500) samples with the thickness of (**d**) 1.6 mm (**e**) 1.8 mm and (**f**) RL values of PVP (214), PVP (333) and PVP (500) samples with the matching thickness of 1.6 mm and 1.8 mm.

Impedance matching is an important factor to the microwave absorbers. If the nanofibers are of good impedance matching properties, more microwave will go into the nanofibers and will be attenuated^32,33^. To synthesize a satisfactory microwave absorber, it is the key to improve the microwave absorption performance or board the effective frequency width. Figure 10 gives the 3D representation of |Z_in_/Z_0_| values of PVP (214), PVP (333) and PVP (500) samples. Obviously, PVP (214) sample owns the most inferior impedance matching properties. For PVP (333) sample, the values of |Z_in_/Z_0_| are almost near to 1, so the effective frequency widths of PVP (333) sample are boarder than PVP (214) sample and PVP (500) sample. And for PVP (500) sample, the values of |Z_in_/Z_0_| can be 1, we obtain the strongest microwave absorption abilities from PVP (500) sample with 2.2 mm, 2.4 mm and 2.6 mm. These results accord to the analysis of Fig. 6. Taken the matching thickness of 1.6 mm and 1.8 mm as examples, it is pleased that the |Z_in_/Z_0_| values of PVP (333) sample and PVP (500) sample are around 1, the RL values are meet the requirements of −10 dB. Particularly, PVP (333) sample gets the strongest microwave absorption abilities and the broadest effective frequency width with the thickness of 1.8 mm.

Conductive networks successfully are changed by different content of PVP. To further compared the conductivity of different content of PVP based carbon nanofibers. The conductivity values are evaluated using on the following formula^2^:

4 $${{\rm{\sigma }}}_{{\rm{AC}}}={{\rm{\varepsilon }}}_{0}{\rm{\varepsilon }}^{\prime\prime} w={{\rm{\varepsilon }}}_{0}{\rm{\varepsilon }}^{\prime\prime} 2\pi f$$

5 $${{\rm{\varepsilon }}}_{0}={10}^{(-9)/36{\rm{\pi }}}$$

**Figure 11:**
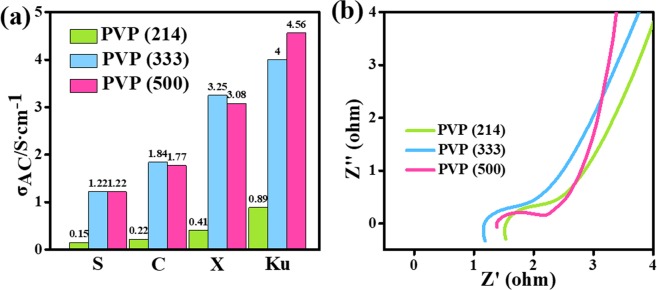
(**a**) Average conductivity values of PVP (214), PVP (333), and PVP (500) samples in S, C, X and Ku band; (**b**) Nyquist plot of PVP (214), PVP (333) and PVP (500).

In Fig. 11, PVP (214) sample shows the lowest average conductivity while the average conductivities of PVP (333) sample and PVP (500) can be greatly improved at S, C, X and Ku frequency range with the increasing content of PVP. The electronic impedance spectrum (EIS) for PVP (214), PVP (333) and PVP (500) were measured to evaluate their conductivity. As can be seen in the Nyquist plot in Fig. 11(b), the theoretical prediction of electrical conductivity in the following order PVP (214) < PVP (333) < PVP (500), indicating the higher conductivity with the increasing content of PVP. In this case, more contact positions of nanofibers will make electrons have multiple paths to transfer, which creates the increased conductivity. The carbon conductive network is fabricated simultaneously.

Table 1 gives some data of similar carbon nanofibers about their microwave absorption performance. In Table 1, we can easily conclude that all kinds of carbon nanofibers show enhanced microwave absorption abilities. Compared with carbon fibers that heap up one by one, nanofibers with some bonded places are more beneficial to make electrons transfer. When this type of carbon nanofiber is put in EM field, electron is likely to transfer with many paths. Hence, among these different carbon nanofibers, our adjustable shaped carbon nanofibers display the minimum RL value and broader effective bandwidth with the thinnest thickness, which implies that it is very potential to be used as lightweight microwave absorber.

**Table 1:** Comparation of some similar nanofibers on their microwave absorption properties.

Sample	RL_mix_ (dB)	Thickness (mm)	Filler loadings (wt%)	Effective bandwidth (<−10 dB)	Ref.
Hollow CNF	−23.0	3.0	33	2.3	^18^
Conductive nanocomposite fibers	−5.9	2.0	—	—	^34^
Porous-carbon-nanotube decorated CNF	−44.5	2.0	20	5.2	^17^
Carbon nanofiber/epoxy	−34.0	2.1	8	3.6	^35^
Porous CNF	−31.0	2.3	6	—	^11^
CNF with adjustable shape	−51.3	1.8	20	5.1	This work

## Conclusion

Carbon nanofibers with adjustable nanostructure were gained by electrospinning method. When calcined at the same temperature (800 °C), the PVP content could be the main reason that influence the microwave absorption abilities. The increased adhesive contacts of nanofibers create the more paths for electrons to transfer and can reduce the effect of air dielectric. Furthermore, appropriate impedance matching is also responsible for the excellent microwave absorption abilities. In detail, the minimum RL value is −51.3 dB at 15.2 GHz and the maximum effective absorption frequency width (<−10 dB) is 5.1 GHz both with 1.8 mm. Our work may be helpful for the exploitation of lightweight microwave absorbents in near future.

## Method

### Materials

All chemicals and reagents are supplied by business supporters and they are all without pretreated. These chemicals are polyacrylonitrile (PAN, M_w_ = 150 000) and polyvinylpyrrolidone (PVP, M_w_ = 5 800). *N*, *N*-dimethylformamide (DMF) is needed as well.

### Synthesis of carbon nanofibers

Carbon nanofibers were obtained by electrospinning technology and following high-temperature carbonization. First, 0.5 g of PAN was added into 5 mL DMF with continuous magnetic stirring. Then, a certain amount of PVP was also introduced to this system with stirring for 24 h to achieve a transparent and syrupy liquid. The mass ratio of PAN and PVP is 7:3, 6:4 and 5:5, respectively. The precursor nanofibers are called PAN/PVP-7/3, PAN/PVP-6/4 and PAN/PVP-5/5, respectively. Second, this liquid was sucked into a 5 mL plastic syringe equipped with a stainless needle. The next process was the electrospinning. Specifically, the voltage parameters were 20 kV, the collection distance was 15 cm and the pushing speed was 0.5 mL/h. After that, the precursor PAN/PVP nanofibers were dried in a vacuum oven for a day and the PAN/PVP nanofibers were calcined at 800 °C for 3 h surrounded with N_2_ atmosphere. Herein, the average rate was of 2 °C per minute. And the carbon nanofibers are marked as PVP (214), PVP (333) and PVP (500), respectively. When these PAN/PVP nanofibers were heated at 200 °C, 400 °C and 600 °C, these samples are named as PVP (214–200), PVP (333–200), PVP (500–200), PVP (214–400), PVP (333–400), PVP (500–400), PVP (214–600), PVP (333–600) and PVP (500–600), respectively.

### Characterization

The crystal structure was measured by X-ray diffraction (XRD) under Cu K*α* radiation. The SEM (Hitachi-S4800) as well as TEM (Tecnai G2 F30) were used to observe the microtopography of these nanofibers. A vector network analyzer (Agilent, N5244A) was applied to test the electromagnetic parameters in the range of 2–18 GHz. The samples (20% filler ratio) and paraffin (80% filler ratio) were crushed into a cylinder. The inner diameter and the outer diameter were 3.0 mm and 7.0 mm. When changing the filler ratio, the samples (30%, 40% and 50% filler ratio) are called as PVP (214, 30), PVP (333, 30), PVP (500, 30), PVP (214, 40), PVP (333, 40), PVP (500, 40), PVP (214, 50), PVP (333, 50) and PVP (500, 50), respectively.
